# Enhanced Reusability of Immobilized T7 DNA Polymerase in Multi-Cycle Exonuclease Reactions on Gold-Coated SAM Biosensor Platforms

**DOI:** 10.3390/bios16010037

**Published:** 2026-01-03

**Authors:** Julija Sarvutiene, Deivis Plausinaitis, Vytautas Bucinskas, Simonas Ramanavicius, Alma Rucinskiene, Arunas Ramanavicius, Urte Prentice

**Affiliations:** 1 State Research Institute Center for Physical Sciences and Technology, Sauletekio Av. 3, LT-10257 Vilnius, Lithuaniaalma.rucinskiene@ftmc.lt (A.R.); arunas.ramanavicius@chf.vu.lt (A.R.); 2 Department of Physical Chemistry, Faculty of Chemistry and Geoscience, Institute of Chemistry, Vilnius University, Naugarduko St. 24, LT-03225 Vilnius, Lithuania; 3 Department of Mechatronics, Robotics and Digital Manufacturing, Faculty of Mechanics, Vilnius Gediminas Technical University, Plytines St. 25, LT-10105 Vilnius, Lithuania; 4 State Research Institute Centre for Innovative Medicine, Santariskiu St. 5, LT-08410 Vilnius, Lithuania

**Keywords:** biosensor, DNA-sensor, DNA polymerase, immobilization, materials, reuse

## Abstract

The reusability of enzymes is a fundamental aspect of sustainable biotechnology and the development of biosensors. This study presents one of the first quantitative evaluations of DNA polymerase reusability by utilizing integrated quartz crystal microbalance (QCM) kinetics and real-time monitoring of exonuclease activity. The results showed that immobilized T7 DNA polymerase retained approximately 50% of its initial activity after three 90-min cycles and around 20% after five cycles. Significantly lower activities were observed for shorter, 45-min cycles. This indicates an unexpected time-dependent enhancement in stability for longer reaction times. The findings suggest a promising trend in enzyme stability and reusability, establishing a quantitative relationship between reaction duration and enzyme performance. This relationship offers a scalable pathway for the regeneration of biosensors and for sustainable enzymatic catalysis. Additionally, the work provides a transferable framework that can be applied to other DNA-processing enzymes, which supports long-term biosensor performance and industrial biocatalysis. The demonstrated approach offers a transferable and scalable methodology for the development of reusable polymerase-based biosensors and sustainable biocatalytic systems.

## 1. Introduction

**Figure 1:**
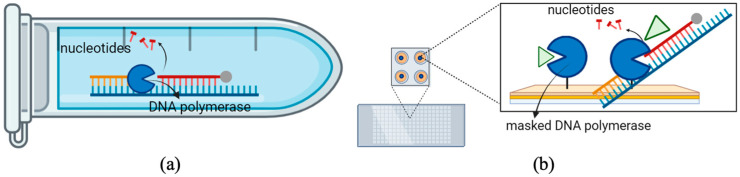
Schematic representation of enzymatic reaction systems. (**a**) In a conventional solution-based assay, DNA polymerase catalyzes the exonuclease reaction, sequentially removing nucleotides from a DNA strand. The reaction mixture, containing the enzyme, DNA fragments, and nucleotide products, is typically discarded after a single use, resulting in higher reagent consumption and limited reusability. (**b**) In contrast, the biosensor-based configuration employs a masked DNA polymerase (green triangle) immobilized on a solid support, such as a gold nanosurface or microarray platform. “Masked” refers to transient active-site occupancy by a double-stranded DNA fragment before the immobilization. The DNA fragment temporarily occupies the 3′→5′ exonuclease site, preventing premature hydrolysis or nonspecific interaction with the SAM surface. The masking effect is sequence-independent. After immobilization and rinsing, the DNA dissociates, restoring the whole active enzyme population.

Enzymes play a central role in green and sustainable biotechnology due to their catalytic efficiency and selectivity. However, their reuse is often limited by the application of dissolved enzymes. To enhance enzyme reusability, enzymes can be immobilized on various support materials. Immobilization on solid supports reduces denaturation and detachment, thereby enhancing enzyme stability. Therefore, immobilized enzymes can be used in repeated operations, thereby improving process efficiency, and reducing environmental impact. Among immobilization techniques (Figure 1b), self-assembled monolayers (SAMs) on gold surfaces enable strong covalent coupling and oriented attachment of immobilized molecules, offering excellent control over enzyme density and activity [1,2]. These platforms are ideal for biosensing applications because they allow label-free, real-time monitoring of binding and catalytic processes.

A study of low-molecular-weight compounds, such as glucose oxidase immobilized on chitosan beads, demonstrated that the enzyme retained 80% of its catalytic activity after ten consecutive operational cycles. In contrast, the free enzyme lost most of its catalytic activity after the same number of cycles [3]. Reusing immobilized enzymes improves efficiency, reduces environmental impact without sacrificing performance, and decreases the cost per reaction cycle. For example, the hydrolysis of penicillin G using immobilized penicillin acylase demonstrated a half-life of 1500 h, whereas the free enzyme had a half-life of only 50 h [4]. The initial activity of free lipase was higher than its immobilized form, with the free enzyme retaining only 30% of its activity after ten cycles. In comparison, the immobilized enzyme maintains 70% of its activity. Free (liquid) enzymes often exhibit greater initial catalytic activity because they are not limited by a support matrix. However, immobilized enzymes demonstrate enhanced stability, allowing for consistent catalytic performance over extended periods [5]. Immobilized enzymes could be utilized in various applications, including industrial biocatalysis, pharmaceuticals, food processing, biofuel production, wastewater treatment, microarray technology, and the development of biosensors [6,7,8]. Furthermore, enzyme-based biosensors are being created to provide precise and reliable analyses for medical diagnostics, environmental monitoring, and food safety testing [9].

High-molecular-weight compounds, such as T7 DNA polymerase, encoded by bacteriophage T7, are highly processive and accurate enzymes widely used in DNA sequencing and molecular biology applications [10,11]. Its fidelity arises from the coordinated action of the polymerase and intrinsic 3′→5′ exonuclease proofreading activities, which together ensure accurate DNA synthesis [12,13]. In addition to proofreading during replication, the 3′→5′ exonuclease domain of T7 DNA polymerase is fully capable of hydrolyzing single-stranded and double-stranded DNA substrates, even in the absence of a primer–template junction. This property enables efficient degradation of blunt-ended dsDNA fragments, such as those used in this study, consistent with the complete digestion observed during the first 90-min reaction cycle. Although T7 DNA polymerase is unsuitable for high-temperature PCR due to its thermal instability, it is ideal for isothermal DNA processing at moderate temperatures [14]. While other DNA polymerases, such as Taq, T4, and Phi29, have been immobilized for amplification or replication studies, most have been tested under single-use conditions without quantitative evaluation of reusability. Unlike previous studies that focused on single-use assays, this work quantitatively correlates enzyme reuse performance with surface immobilization kinetics under controlled QCM monitoring [15].

Immobilization of high-molecular-weight and multimeric enzymes requires strategies that enhance stability without compromising flexibility and activity [16,17]. Covalent coupling via EDC/NHS (EDC (N-(3-dimethylaminopropyl)-N′-ethylcarbodiimide; NHS (N-hydroxysuccinimide)) activation on carboxyl-terminated SAMs or nanoparticles provides strong and stable attachment. At the same time, multipoint binding on epoxy- or glyoxyl-activated supports distributes mechanical stress and improves rigidity. For instance, β-galactosidase immobilized on glyoxyl-agarose retained its catalytic function five times longer than when it was immobilized through single-site coupling [18]. Affinity-based approaches, such as His-tag/Ni^2+^-NTA or biotin-streptavidin systems, ensure proper enzyme orientation, minimize steric hindrance, and allow reversible reuse, with His-tagged dehydrogenases retaining over 85% activity after 10 cycles. Entrapment in sol–gel or mesoporous matrices provides a hydrated, protective environment that maintains structural integrity, while nanostructured supports, such as mesoporous silica or graphene frameworks, enhance substrate diffusion and turnover. Laccase immobilized on 3D graphene maintained more than 70% of its activity after 15 reuse cycles [19,20]. Overall, integrating orientation control, covalent stabilization, and nanostructured supports enables efficient immobilization of HMW enzymes, balancing mechanical stability with high catalytic performance and long-term reusability.

The present work bridges this gap by integrating real-time QCM monitoring with kinetic modeling to provide a quantitative description of enzyme performance under biosensor-relevant conditions. In this context, surface regeneration and enzyme substitution were deliberately excluded to isolate the intrinsic operational stability of immobilized T7 DNA polymerases. The primary objective of this study is to evaluate the stability and reusability of immobilized multimeric masked T7 DNA polymerase under varying reaction conditions through detailed kinetic and enzymatic activity analyses. We selected T7 DNA polymerase because it has high proofreading activity and operates optimally at room temperature (approximately 25 °C), making it particularly suitable for practical applications that do not require controlled high-temperature environments. In contrast, other high-molecular-weight enzymes typically require higher operational temperatures (above 37 °C), making them less suitable for such setups. In addition, T7 DNA polymerase is a well-characterized, highly processive enzyme with both polymerase and exonuclease (proofreading) domains, enabling precise monitoring of substrate turnover and degradation on the QCM surface. Its monomeric catalytic core and relatively stable tertiary structure enable immobilization without substantial loss of activity. Moreover, the enzyme’s broad usage in molecular biology and well-documented kinetic parameters make it an ideal model system for establishing a reliable QCM-based platform to study enzymatic reactions at solid–liquid interfaces. This work establishes a direct relationship between kinetic adsorption models and the practical reusability of covalently immobilized T7 DNA polymerase on SAMs integrated with quartz crystal microbalance (QCM) sensors. The immobilization of masked T7 DNA polymerase on a gold QSense^®^ flow-through surface was investigated to enable its reuse in hydrolysis reactions involving high concentrations of DNA fragments. Building on our previous work, in which we developed and optimized covalent immobilization protocols for T7 DNA polymerase on SAM-functionalized gold electrodes while retaining its exonuclease activity [21], this study extends the approach to assess the enzyme’s stability and functional reusability during multiple hydrolysis cycles. The findings demonstrate that this immobilization strategy not only enhances the operational stability of T7 DNA polymerase but also provides a cost-effective and sustainable platform for repeated use in DNA processing and biosensing applications. The scope of this study was deliberately focused on a single enzyme–SAM configuration to ensure experimental reproducibility and isolate intrinsic reusability parameters without regeneration or multi-enzyme interference

## 2. Materials and Methods

12-Mercaptododecanoic acid (HS(CH_2_)_11_COOH; 12-MUA, CAS 82001-53-4, 96% purity), 6-mercapto-1-hexanol (HS(CH_2_)_6_OH; 6-MCH, CAS 1633-78-9, 97% purity), N-hydroxysuccinimide (NHS, CAS 6066-82-6, 98% purity), N-(3-dimethylaminopropyl)-N′-ethylcarbodiimide hydrochloride (EDC, CAS 25952-53-8, 98% purity), and ethanol (99.5%) were obtained from Sigma-Aldrich (Steinheim, Germany).

Agarose (R0491), Tris–acetate–EDTA (TAE) buffer (50×; B49), phosphate-buffered saline (PBS, 10×, pH 7.4; AM9625), nuclease-free water (AM9906), a low-DNA-mass ladder (10068013), T7 DNA polymerase (10 U/µL, EP0081), and the corresponding 10× reaction buffer were supplied by Thermo Scientific Baltics, Vilnius, Lithuania.

A 1% ethidium bromide solution (2218.1) for agarose gel staining was purchased from Carl Roth (Karlsruhe, Germany). Concentrated sulfuric acid (H_2_SO_4_, >96.0%, CAS 7664-93-9) was sourced from Lachner (Neratovice, Czech Republic). Desalted, unmodified DNA oligonucleotides and 3′-thiol-modified C6 S–S DNA oligonucleotides, as well as DL-dithiothreitol (DTT, CAS 3483-12-3), were obtained from Sigma-Aldrich and used as received without additional purification.

Gold-coated QCM electrodes were modified with mixed SAMs of 12-MUA (11-mercaptoundecanoic acid) and 6-MCH (6-mercapto-1-hexanol) (6:4 molar ratio). Carboxyl groups were activated using EDC/NHS chemistry, followed by immobilization of masked T7 DNA polymerase (EP0081 Thermo Scientific) under controlled flow (0.69 µM, 0.1 mL/min, 2 h). Before immobilization, a transient substrate-occupancy masking step was applied as reported in our previous studies [21]. This reversible blocking prevents premature exonucleolytic activity during covalent attachment. After immobilization on the SAM surface, unbound DNA was removed by rinsing with phosphate buffer, leading to spontaneous unmasking and the restoration of enzyme activity. Reusability experiments were conducted using 1 ng of double-stranded DNA substrate in buffer at room temperature for 45 and 90 min. Each reuse experiment was performed in triplicate, and mean values with standard deviations are reported. Between cycles, the surface was rinsed with phosphate buffer (pH 7.4) without chemical regeneration to maintain identical boundary conditions. Hydrolysis efficiency was assessed over five consecutive reaction cycles using 2% agarose gel electrophoresis with SYBR Safe staining. For each experimental run (45 min or 90 min), all samples—including the zero-time control and the five reuse-cycle reactions—were generated from a single reaction master mix prepared immediately before loading. This ensured identical DNA and buffer composition across all lanes within each gel. Variability between lanes, therefore, reflects only pipetting/loading differences and not differences in reaction setup. Band intensity was quantified with TotalLab 2.0. software and converted into relative activity values. The Sauerbrey equation, which is based on the correlation between changes in resonance frequency and mass of the QCM-chip, was applied for the determination of DNA-sequence formation. Three independent measurements were performed for each experimental point.

## 3. Results

### 3.1. Characterization of Free Enzyme Study

**Figure 2:**
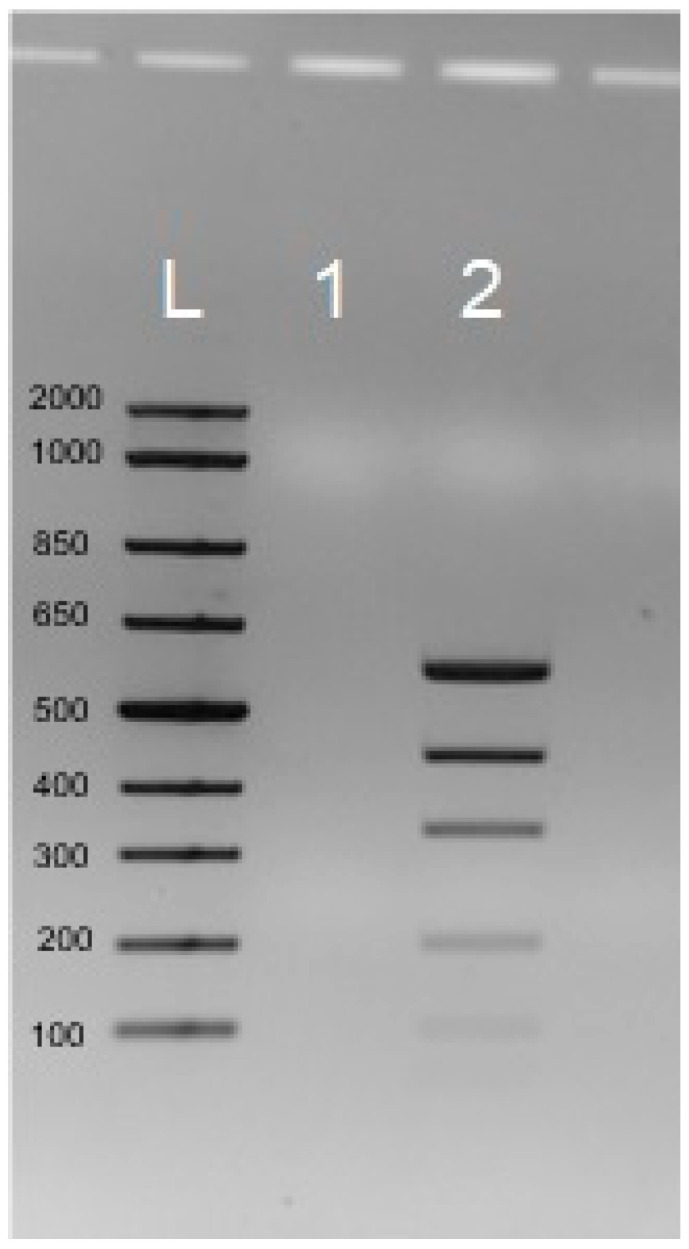
1 µg of DNA is hydrolyzed over 21 h with 12.5 U of T7 DNA polymerase. L—DNA ladder; 1—1ug of Low DNA Mass Ladder after the hydrolysis with the T7 DNA polymerase over 21 h; 2—1ug of Low DNA Mass Ladder as a control reaction without the T7 DNA polymerase for over 21 h. L indicates the molecular-weight DNA ladder (100–2000 bp). Lane 1: Low DNA Mass Ladder after hydrolysis with T7 DNA polymerase for 21 h. Lane 2: control reaction without enzyme. All samples were run on 2% TAE agarose gels stained with SYBR Safe.

This part of the study examined the use of 1 µg of Low DNA Mass Ladder as a double-stranded DNA (dsDNA) substrate following a 21-h reaction in the T7 DNA polymerase buffer (400 mM Tris-HCl (pH 7.5 at 25 °C), 100 mM MgCl_2_, and 10 mM DTT). The results confirmed the complete hydrolysis of the 1 µg Low DNA Mass Ladder over the 21-h in vitro reaction, using 12.5 U of T7 DNA polymerase. Results were obtained for both responses, with and without the enzyme, as shown in Figure 2.

**Figure 3:**
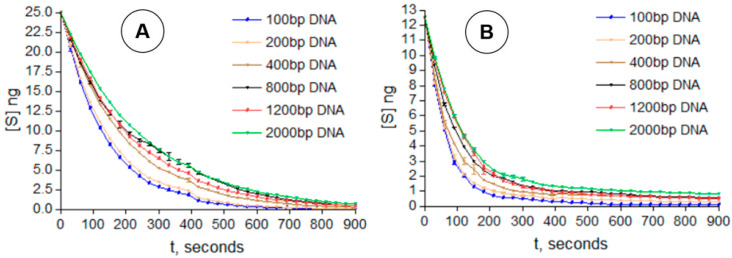
Hydrolysis of various DNA fragment lengths using primary substrate concentration of 25 ng (**A**) and 12.5 ng (**B**) in the T7 DNA polymerase 3′→5′ exonuclease activity reaction.

To study the behavior of free T7 DNA polymerase, the hydrolysis of DNA fragments of varying lengths was measured at different concentrations of DNA substrate. Various lengths of DNA were extracted from a Low DNA Mass Ladder using agarose gel electrophoresis to prepare specific-length fragments for testing exonuclease activity. A 2% TAE (Tris-Acetate-EDTA buffer) agarose gel was used for separation, and target bands (100–2000 bp) were visualized under UV light. These bands were extracted and purified using a GeneJET Gel Extraction Kit. The absorbance of each fragment (100 bp, 200 bp, 400 bp, 800 bp, 1200 bp, and 2000 bp) was measured at 260 nm with a Nanodrop spectrophotometer to determine concentration and assess purity by calculating the A260/A280 ratio. The final concentration of each fragment was set at 0.25 mg/mL. DNA concentrations were adjusted to 25 ng and 12.5 ng per reaction, and T7 DNA polymerase was included at a concentration of 10 U. The reactions were conducted in a 96-well microplate with standard buffer conditions (400 mM Tris-HCl, pH 7.5; 100 mM MgCl2; 10 mM DTT) and Mg^2+^ as a cofactor for nucleophilic activity. During hydrolysis, the 3′ terminal nucleotide aligns with the enzyme’s active site, where two Mg^2+^ ions facilitate the reaction. One ion activates a water molecule for nucleophilic attack, while the other stabilizes the leaving phosphate’s negative charge. This hydrolysis releases a deoxynucleotide monophosphate (dNMP). The hydrolysis of different DNA length fragments during the enzymatic reaction was monitored by measuring absorbance at 260 nm at regular intervals (every 30 s for 1 h). These absorbance values were converted into the remaining DNA concentration at each time point (Figure 3). To investigate the kinetics of DNA hydrolysis, the DNA hydrolysis reaction was initially analyzed while using a first-order kinetic model.

Control experiments using non-immobilized (free) T7 DNA polymerase were conducted under standard buffer and substrate conditions. The free enzyme completely hydrolyzed 25 ng and 12.5 ng of dsDNA within 15 min, confirming that the substrate length did not limit the conversion rate.

### 3.2. Characterization of Enzyme-Based Monolayer Study

**Figure 4:**
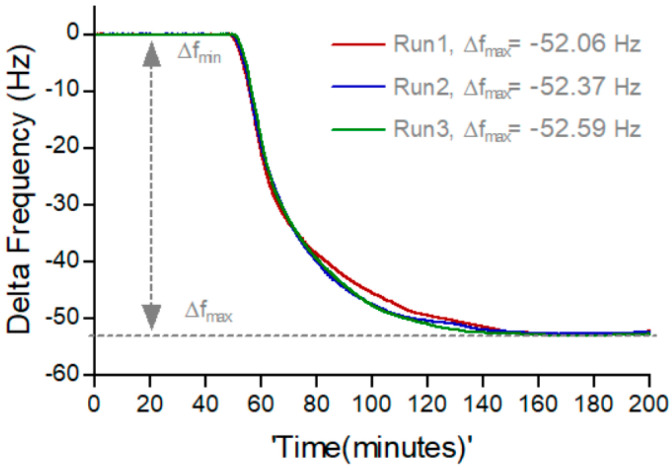
Representative QCM profiles showing the immobilization and saturation behavior of masked T7 DNA polymerase on a gold surface modified with a 6:4 ratio of MUA: MCH SAM. The resonance frequency shifts (*f*_max_) were consistent across three independent experiments, indicating high reproducibility and stable enzyme attachment to the functionalized surface. Real-time QCM frequency shifts (Δf) recorded during immobilization of masked T7 DNA polymerase on a 6:4 MUA:MCH SAM. Measurements were acquired under continuous flow (0.1 mL/min). The rapid decline corresponds to initial enzyme adsorption, followed by covalent attachment and saturation.

Gold-coated QCM electrodes were functionalized with a 6:4 ratio of 11-mercaptoundecanoic acid (MUA) to 6-mercapto-1-hexanol (MCH) SAM, previously identified as optimal for T7 DNA polymerase immobilization [21]. Following NHS/EDC(N-Hydroxysuccinimide/N-(3-Dimethylaminopropyl)-N′-Ethylcarbodiimide Hydrochloride) activation, the carboxyl groups on the SAM surface were coupled with masked T7 DNA polymerase under controlled flow conditions (0.69 µM, 0.1 mL/min, 2 h). Real-time QCM traces displayed a rapid frequency decline during the first 40 min, corresponding to the active enzyme immobilization, followed by a slower stabilization phase that reached a plateau near 160 min (Figure 4). This two-phase response indicates rapid surface adsorption initially, followed by the gradual completion of covalent coupling reactions, leading to a stable enzyme monolayer.

Our study assumes that the immobilization of masked T7 DNA polymerase is primarily governed by kinetic factors, meaning that the rate of molecular attachment to the sensor surface predominantly determines the formation of the Au/12-MUA+6-MCH/enzyme layer. This layer was considered to be rigidly and firmly bound to the sensor surface, thereby ensuring measurement stability. Under these conditions, viscoelastic effects, which describe time-dependent deformation of soft or flexible layers, were neglected. Consequently, the observed frequency shifts were interpreted using the Sauerbrey equation, which establishes a direct relationship between the change in resonance frequency and the mass variation on the sensor surface [22].

### 3.3. Characterization of Enzyme-Based Sensor Usage

**Figure 5:**
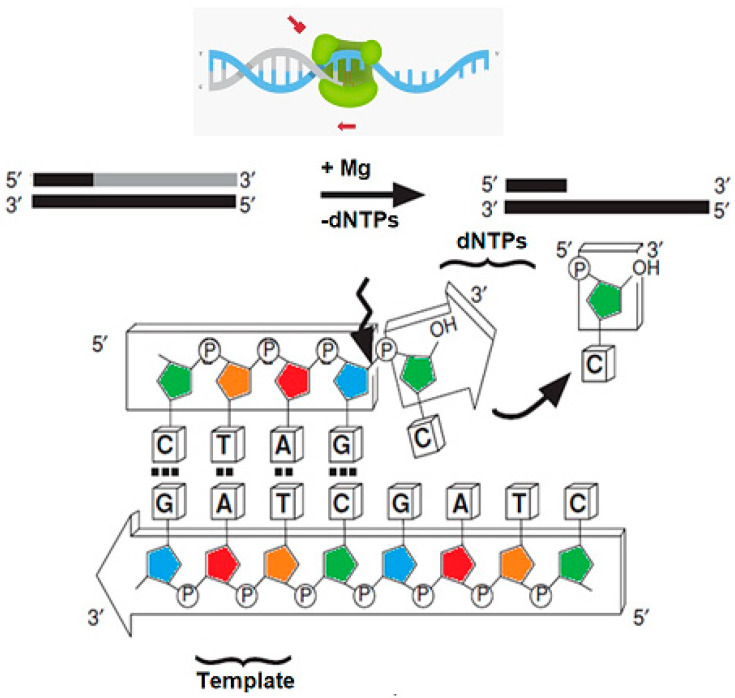
Schematic representation of exonuclease activity. The exonuclease activity of T7 DNA polymerase is used to remove a mismatched or terminal nucleotide from the DNA strand. The enzyme removes nucleotides one by one from the 3′ end of the DNA strand, moving in the 3′→5′ direction.

The Low DNA Mass Ladder consists of double-stranded DNA fragments ranging from 100 bp to 2000 bp, each possessing blunt ends where both strands terminate at the same nucleotide, resulting in no overhangs [23,24]. Blunt-ended DNA fragments are generally more structurally stable but may exhibit lower efficiency in specific enzymatic reactions, such as ligation. T7 DNA polymerase binds to these blunt-ended DNA fragments, which serve as suitable substrates for its 3′→5′ exonuclease activity. This enzymatic function progressively removes nucleotides from the 3′ terminus of the DNA strand [25]. This proofreading domain is responsible for substrate degradation observed in the immobilized system. During this process, the enzyme cleaves the phosphodiester bonds between adjacent nucleotides, releasing mononucleotides sequentially from the 3′ end until it either dissociates from the DNA (Figure 5) or the fragment becomes fully hydrolyzed [26,27].

**Figure 6:**
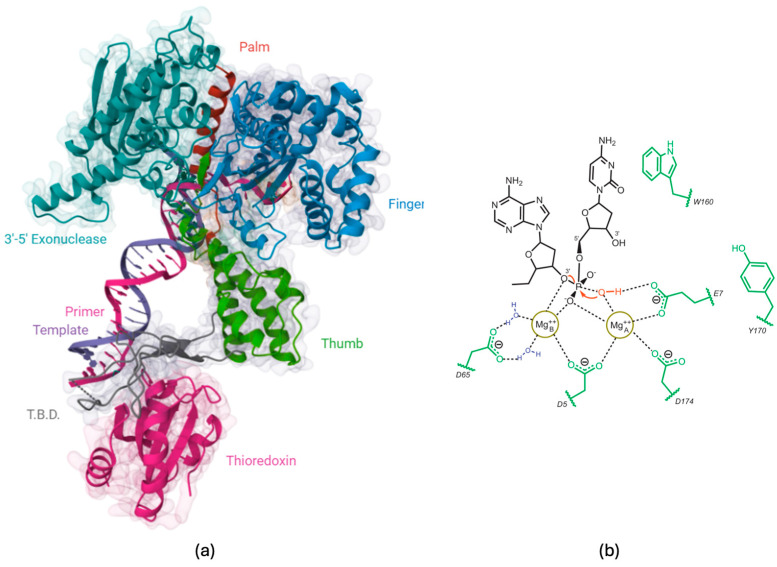
Structural and catalytic features of T7 DNA polymerase. (**a**) Three-dimensional structure of T7 DNA polymerase bound to DNA and thioredoxin, showing the major functional domains: palm (red), finger (blue), thumb (green), 3′→5′ exonuclease (teal), thioredoxin-binding domain (T.B.D.) (gray), and thioredoxin (magenta). The primer (purple) and template (black) DNA strands are displayed within the enzyme’s active site. (**b**) Schematic representation of the 3′→5′ exonuclease catalytic mechanism. Two Mg^2+^ ions (yellow circles) coordinate with key residues (D5, D65, D174, E7, Y170, and W160) to stabilize the scissile phosphate group and activate a water molecule for nucleophilic attack, resulting in phosphodiester-bond cleavage and sequential nucleotide excision. Arrows represent conformational changes, illustrating that domains undergo dynamic movements rather than remaining in a static configuration.

These polymerases favor correct (canonical) base pairing over mismatches by approximately 100,000-fold, and their fidelity is further enhanced by a 3′→5′ proofreading exonuclease that removes misincorporated bases. Although proofreading plays a crucial role in maintaining genome stability, it has been studied less extensively than the fidelity mechanisms of the polymerase active site. In this study, substrate preference was qualitatively evaluated by comparing the kinetics of fragments of different lengths. Figure 6b illustrates the catalytic mechanism of T7 DNA polymerase’s exonuclease activity, highlighting the metal ion-dependent cleavage of the phosphodiester bond. The schematic shows a nucleotide residue with its phosphate backbone (black), and the cleavage site—where bond hydrolysis occurs—is marked in red to indicate the scissile phosphate targeted during exonucleolytic cleavage. Two magnesium ions (Mg^2+^ A and Mg^2+^ B, yellow circles) participate in a two-metal-ion catalysis mechanism, a hallmark of many exonucleases and polymerases. These ions stabilize the negative impact on the phosphate group, promote nucleophilic attack, and facilitate the release of the leaving group. The catalytic residues Asp5, Asp65, Asp174, and Glu7 coordinate the metal ions, ensuring a proper catalytic geometry. Tyrosine (Tyr170) and tryptophan (Trp160) residues likely contribute to substrate positioning and stabilization during catalysis. The metal ions activate a water molecule (blue), generating a hydroxide ions (red) that performs a nucleophilic attack on the phosphodiester bond, leading to bond cleavage. This hydrolytic reaction releases the terminal nucleotide, thereby enabling efficient exonucleolytic proofreading by T7 DNA polymerase.

**Figure 7:**
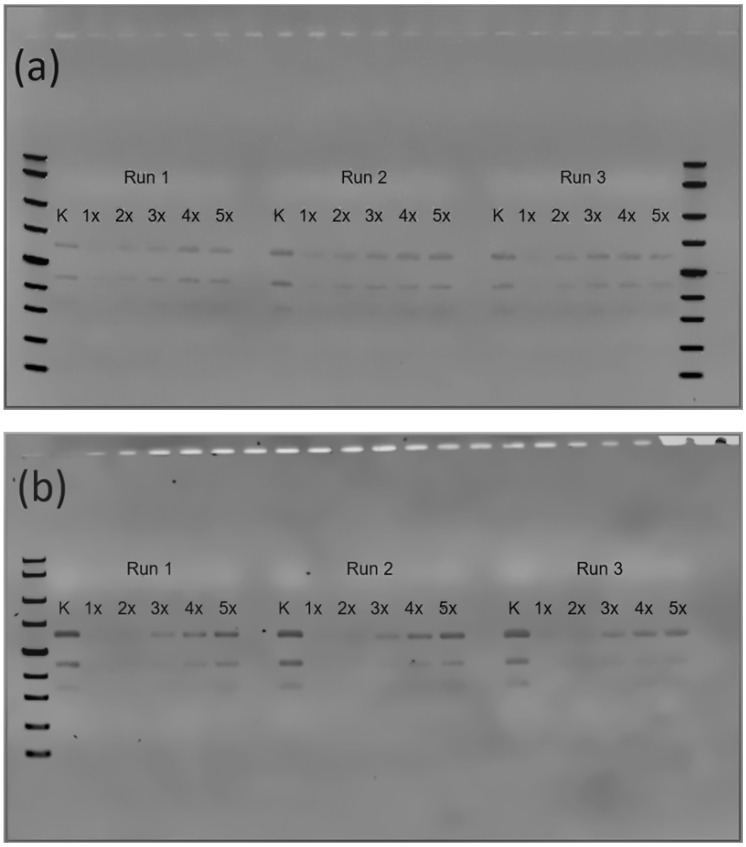
Reusability study of the Au/12-MUA+6-MCH/T7 DNA polymerase monolayer analyzed by agarose gel electrophoresis. Agarose gels (2% TAE buffer with SYBR Safe stain) show the exonuclease activity of immobilized, masked T7 DNA polymerase over five consecutive reaction cycles (lanes 1–5). K indicates the substrate-only control reaction. DNA ladder (L) with indicated size markers (100–2000 bp) is included for reference. Reactions were performed at room temperature for (**a**) 45 min and (**b**) 90 min. Band intensity was quantified using optical band volume analysis. Lanes include a molecular-weight DNA ladder with visible size markers (100–2000 bp) indicated on the gel.

In this study, the described process was applied to evaluate the functionality and reusability of immobilized masked T7 DNA polymerase. QCM measurements combined with kinetic modeling confirmed that the Au/12-MUA+6-MCH/T7 DNA polymerase monolayer remained stable, reaching an enzyme saturation level of 427.95 ± 0.60 ng. The substrate concentration was carefully selected based on the processivity of T7 DNA polymerase to ensure optimal reaction conditions [28,29,30]. During this study, we observed that a 15–30-min reaction at room temperature with a buffer solution containing magnesium ions and 1 ng of a double-stranded DNA fragment did not yield clearly detectable exonuclease activity from masked T7 DNA polymerase. Thus, the reaction time (up to 45 min) was optimized to make the system more suitable for rapid and effective detection at room temperature (Figure 7).

The reusability, stability, and exonuclease efficiency of immobilized masked T7 DNA polymerase were evaluated using 2% TAE agarose gel electrophoresis and optical band-volume analysis. The experimental setup included control lanes (K), representing reactions without enzyme, and reuse cycles (1–5), which enabled the assessment of exonuclease activity and the progressive hydrolysis of double-stranded DNA fragments across consecutive runs. The 2% TAE agarose gel electrophoresis images display gradual changes in DNA band intensity, reflecting a decrease in substrate hydrolysis efficiency after multiple reaction cycles. Reactions were performed for 45 min (a) and 90 min (b) to simulate different substrate processing conditions. The electrophoretic data were quantified using the TotalLab system by converting DNA band intensity into optical band volume, providing an objective measure of enzyme reusability and relative activity. Further analysis aims to correlate optical band volume with exonuclease performance across reuse cycles, particularly when activity declines over time. All samples within each experiment, including the K (control) lane and all reuse-cycle lanes, were prepared from the same reaction master mix. Therefore, the initial DNA concentration and reaction composition were identical across all samples within a given experiment. Any minor variation observed between individual bands thus arises solely from pipette-loading variability or gel-to-gel staining differences, rather than from differences in reaction efficiency. The immobilized enzyme retained approximately 50% activity at cycle 3 and 20% at cycle 5 for the 90-min reaction, whereas the 45-min system decreased to 25% and nearly 0%, respectively.

**Figure 8:**
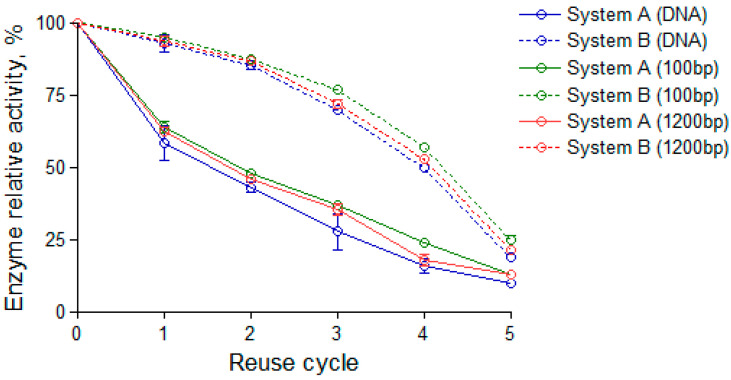
The reuse efficiency of immobilized masked T7 DNA polymerase under two conditions: System A (solid line), a 45-min reaction, and System B (dashed line), a 90-min reaction, with the same concentration of 1 ng of double-stranded DNA fragment. The y-axis shows the relative enzymatic activity (%), while the x-axis represents the number of reuse cycles (0 to 5). In the image, DNA represents the Low DNA Mass Ladder, where 100 bp indicates the hydrolysis of 100 bp DNA length fragments, and 1200 bp indicates the hydrolysis of 1200 bp DNA length fragments.

Error bars represent the standard deviation from three independent replicates, confirming the high reproducibility of the enzymatic response. By the third reuse cycle, the 90-min system retained ~50% of its initial activity (Figure 8), while the 45-min reaction fell below 25%, confirming the time-dependent enhancement of stability. Although fluorescence-based DNA quantification provides a more linear dynamic range, its application was limited in this study because all reactions were performed directly on the QCM surface and collected in minimal elution volumes to preserve identical surface boundary conditions and prevent enzyme desorption. These volumes were insufficient for reliable fluorometric measurements. Future studies will incorporate fluorescence-based assays when sample collection can be decoupled from the surface-bound reaction environment, enabling complementary validation of the gel-derived quantification.

## 4. Discussion

This study successfully established a quantitative, reproducible framework for assessing enzyme reusability by integrating QCM-based kinetic modeling with electrophoretic validation, a method rarely reported for DNA-processing enzymes. Immobilization and reaction duration were identified as the primary determinants of long-term catalytic stability. The results clearly demonstrate that immobilization has a profound impact on reuse efficiency, with reaction duration emerging as a crucial determinant of performance. Specifically, immobilized T7 DNA polymerase exhibited markedly different activity retention at 45 and 90 min, whereas the free enzyme required only 15 min for complete hydrolysis. This demonstrates how surface confinement modifies enzymatic kinetics, restricting enzyme mobility and reducing substrate accessibility, and introduces time-dependent limitations on catalytic efficiency. Our data reveal that reaction duration significantly influences activity retention, primarily by improving substrate turnover rather than by enzyme desorption. The n-order kinetic modeling supports this observation, suggesting non-linear reaction rates resulting from enzyme crowding, limited diffusion, or cooperative effects on the sensor surface. Shorter reactions, such as 45 min, do not allow sufficient substrate–enzyme interaction, leading to incomplete hydrolysis and a more pronounced decline in activity across reuse cycles, likely due to partial denaturation and steric hindrance.

The study was limited to five reuse cycles because extending beyond this range produced unstable enzyme recovery and diffusion profiles under identical surface conditions. In this context, “unstable profiles” refer specifically to QCM baseline drift and increasing noise observed after the fifth cycle, which we attribute to partial relaxation of the SAM-polymerase layer and reduced robustness of the surface-bound enzyme film. This effect is intrinsic to the immobilized configuration: beyond five cycles, the adsorption–desorption equilibrium becomes progressively perturbed, leading to inconsistent mass responses and non-reproducible frequency shifts. For this reason, cycles beyond five were excluded, as they did not meet our reproducibility criteria under identical surface and buffer conditions. These five cycles provided a reliable operational window to evaluate intrinsic catalytic decay and surface kinetics without the confounding effects of regeneration [31]. Surface regeneration was intentionally omitted to isolate intrinsic stability and avoid artifacts from partial enzyme reactivation or structural rearrangement. This design choice aligns with other enzyme stability assessments that limit reuse cycles to eliminate recovery bias. Previous regeneration-based immobilization studies have reported inconsistent recovery rates, ranging from 20% to 60% of the initial activity after EDC/NHS reactivation or alkaline washing, depending on the enzyme density and linker type [18,32]. Such variability complicates direct comparison across immobilization systems.

Despite this focused scope, the study achieved several significant milestones. The results provide direct evidence that immobilization parameters and reaction duration jointly dictate long-term activity retention. This work established a reproducible, quantitative framework that integrates QCM monitoring, kinetic modeling, and electrophoretic validation, thereby forming a baseline for the future development of regenerable multi-enzyme biosensor systems. Comparable improvements in enzyme stability have been demonstrated in co-tethered T7 RNA polymerase and magnetic bead systems, achieving more than 20 transcription cycles with minimal activity loss [33]. Their stability derives from proximity-induced reactivity and reduced enzyme leaching enabled by magnetic scaffolds. However, those studies targeted transcription rather than exonuclease activity. In contrast, this work focuses on DNA hydrolysis, where substrate orientation, steric effects, and reaction duration are decisive. Likewise, Taq DNA polymerase immobilized on SAMs showed partial reusability [34,35], yet lacked the kinetic resolution and direct hydrolysis monitoring achieved in this study. By quantifying enzyme performance across multiple cycles, this study fills a methodological gap. It demonstrates that integrating QCM-based analysis with optical assays enables precise in vitro monitoring of immobilized enzyme dynamics.

Beyond methodological innovation, this research highlights the broader potential of immobilized T7 DNA polymerase as a model for reusable catalytic systems. The enzyme maintained functional stability across cycles, demonstrating the feasibility of developing durable, recyclable catalysts for biotechnology. Such systems are highly relevant for industrial biocatalysis, where immobilization lowers operational costs and enhances process sustainability. Applications span pharmaceuticals (biotransformation and drug synthesis), food processing (flavor enhancement and ingredient stabilization), and biofuel production (biomass-to-sugar conversion) [36]. The immobilization techniques demonstrated here also provide a foundation for the development of biosensors. T7 DNA polymerase and similar enzymes can be integrated into analytical platforms for medical diagnostics, environmental monitoring, and food safety testing, where precision and repeatability are essential. To further optimize performance, future research should refine surface chemistry, linker architecture, and immobilization density while coupling enzyme engineering to enhance selectivity and longevity.

Although the present system is intentionally limited to five cycles without surface regeneration, it achieves high reproducibility and clear mechanistic insight. Future extensions will incorporate regeneration protocols, comparative multi-enzyme studies, and diverse SAM compositions to assess generality and operational scalability. Collectively, these results demonstrate the successful establishment of a quantitative and reproducible framework for enzyme reusability, a key step toward next-generation biosensors and sustainable industrial biocatalysis. The controlled, repeatable performance of the immobilized T7 DNA polymerase demonstrates its suitability for real-time biosensing and flow-through microreactor applications. Overall, this study presents the first validated kinetic–spectroscopic framework for quantifying DNA polymerase reusability, thereby bridging the gap between enzyme immobilization chemistry and biosensor functionality.

## 5. Conclusions

The proposed model can be expanded to other DNA-processing enzymes, enabling a generalized understanding of immobilization dynamics in catalytic and sensing systems. In this work, the enzyme was covalently immobilized on self-assembled monolayer (SAM)-functionalized gold electrodes composed of a 12-MUA/6-MCH mixed monolayer. The immobilized enzyme maintained catalytic activity across five reuse cycles, demonstrating significantly enhanced stability under extended reaction durations. Specifically, a 90-min incubation preserved approximately 50% of the initial activity by the third reuse cycle and about 20% by the fifth. In contrast, the 45-min setup exhibited a sharper decline, reaching 25% and approaching depletion.

These findings highlight the significant impact of reaction time on the operational stability of immobilized enzymes. By combining label-free quartz crystal microbalance (QCM) monitoring with kinetic modeling, this study establishes a quantitative framework for understanding and optimizing enzyme immobilization and reuse. The demonstrated approach offers a cost-effective and sustainable pathway for applications in DNA diagnostics, enzymatic biosensors, and sequencing technologies, where enzyme longevity and reusability are essential for reliable and scalable performance. The established framework provides a foundation for the development of regenerable, multi-enzyme biosensors and sustainable biocatalytic platforms applicable across biotechnology and diagnostic industries.

## Data Availability

Any additional information contained in this document is available upon request from the corresponding author (U.P.).
